# Characterization of an engineered human purine nucleoside phosphorylase fused to an anti-her2/*neu *single chain Fv for use in ADEPT

**DOI:** 10.1186/1756-9966-28-147

**Published:** 2009-12-03

**Authors:** Sepideh Afshar, Tove Olafsen, Anna M Wu, Sherie L Morrison

**Affiliations:** 1 Department of Microbiology, Immunology, and Molecular Genetics, UCLA, 247 BSRB, 615 Charles E. Young East, Los Angeles, 90095, USA; 2 David Geffen School of Medicine Crump Inst, UCLA, 615 Charles E. Young East, BOX 95177, Los Angeles, 90095, USA

## Abstract

**Background:**

Antibody Directed Enzyme Prodrug Therapy (ADEPT) can be used to generate cytotoxic agents at the tumor site. To date non-human enzymes have mainly been utilized in ADEPT. However, these non-human enzymes are immunogenic limiting the number of times that ADEPT can be administered. To overcome the problem of immunogenicity, a fully human enzyme, capable of converting a non-toxic prodrug to cytotoxic drug was developed and joined to a human tumor specific scFv yielding a fully human targeting agent.

**Methods:**

A double mutant of human purine nucleoside phosphorylase (hDM) was developed which unlike the human enzyme can cleave adenosine-based prodrugs. For tumor-specific targeting, hDM was fused to the human anti-HER2/*neu *single chain Fv (scFv), C6 MH3B1. Enzymatic activity of hDM with its natural substrates and prodrugs was determined using spectrophotomeric approaches. A cell proliferation assay was used to assess the cytotoxicity generated following conversion of prodrug to drug as a result of enzymatic activity of hDM. Affinity of the targeting scFv, C6 MH3B1 fused to hDM to Her2/*neu *was confirmed using affinity chromatography, surface plasmon resonance, and flow-cytometry.

**Results:**

*In vitro *hDM-C6 MH3B1 binds specifically to HER2/*neu *expressing tumor cells and localizes hDM to tumor cells, where the enzymatic activity of hDM-C6 MH3B1, but not the wild type enzyme, results in phosphorolysis of the prodrug, 2-fluoro-2'-deoxyadenosine to the cytotoxic drug 2-fluoroadenine (F-Ade) causing inhibition of tumor cell proliferation. Significantly, the toxic small drug diffuses through the cell membrane of HER2/*neu *expressing cells as well as cells that lack the expression of HER2/*neu*, causing a bystander effect. F-Ade is toxic to cells irrespective of their growth rate; therefore, both the slowly dividing tumor cells and the non-dividing neighboring stromal cells that support tumor growth should be killed. Analysis of potential novel MHCII binding peptides resulting from fusion of hDM to C6 MH3B1 and the two mutations in hDM, and of the structure of hDM compared to the wild-type enzyme suggests that hDM-C6 MH3B1 should exhibit minimal immunogenicity in humans.

**Conclusion:**

hDM-C6 MH3B1 constitutes a novel human based protein that addresses some of the limitations of ADEPT that currently preclude its successful use in the clinic.

## Background

Specific delivery of therapeutic drugs to tumor cells has been a major focus of cancer therapy. One approach to specific drug delivery has been the use of Antibody Dependent Enzyme Prodrug Therapy (ADEPT) in which an enzyme is joined to a tumor specific antibody which localizes the enzyme in the vicinity of the tumor. A relatively non-toxic prodrug, which is a substrate for the enzyme, is then administered and converted to a cytotoxic drug at the tumor site where the enzyme is localized, resulting in tumor cell death [1-4]. For ADEPT to be effective, the prodrug must be cleaved to a cytotoxic agent only by the administered enzyme [4]. Therefore, endogenously expressed human enzymes cannot be utilized for ADEPT, since the prodrug will be converted to a cytotoxic drug not only in the vicinity of tumor, but also at sites where endogenous enzyme is expressed causing systemic toxicity. On the other hand, if a non-human enzyme is used, it will be immunogenic, preventing multiple administrations [2].

One strategy for achieving effective ADEPT is to change the substrate specificity of a human enzyme such that it can cleave prodrugs that are not substrates of wild type enzyme. Recently, we have reported a mutated human purine nucleoside phosphorylase that is capable of utilizing adenosine-based prodrugs as substrate [5]. The endogenously expressed human purine nucleoside phosphorylase (hPNP) cleaves 6-oxo purines to their corresponding free base and ribose-1-phosphate, but does not use adenosine or adenosine-based prodrugs [5,6]. However, following two mutations (Glu201Gln:Asn243Asp) in the purine binding pocket of hPNP the resulting enzyme (hDM) effectively cleaves adenosine-based prodrugs including 2-fluoro-2'-deoxyadenosine (F-dAdo), Cladribine, and 2-fluoroadenosine to their corresponding cytotoxic base [5]. When the activity of hDM was tested *in vitro*, generation of the toxic metabolite 2-fluoroadenine (F-Ade) due to phosphorolysis of F-dAdo resulted in inhibition of cell proliferation and apoptosis of tumor cells [5]. Therefore, hDM-F-dAdo constitutes an attractive enzyme-prodrug combination for use in ADEPT.

We now report the further development of hDM for use in ADEPT. To localize hDM to tumors, it was fused at its C-terminus to an anti-HER2/*neu *single chain Fv (scFv), C6 MH3B1 via a rigid α-helical linker. C6 MH3B1 is the result of affinity maturation of the scFv C6.5 isolated from a fully human non-immune phage library [7] and exhibits high specificity, affinity, and most importantly a slow dissociation rate from the tumor associated antigen, HER2/*neu *[7]. The fusion protein, hDM-C6 MH3B1 forms an active trimer capable of cleaving F-dAdo to F-Ade in a dose-dependent manner with kinetic parameters comparable to those previously reported [5]. *In vitro *hDM-C6 MH3B1 localizes to tumor cells and its cleavage of F-dAdo results in tumor cell death. The F-Ade generated will also inhibit the proliferation of neighboring tumor cells that lack expression of the tumor antigen, the so called "bystander effect". Moreover, we showed that F-Ade is as toxic to slowly growing and non-proliferating cells as it is to rapidly dividing tumor cells. The binding affinity of hDM-C6 MH3B1 for HER2/*neu *was found to be similar to that of the single chain C6 MH3B1, with hDM-C6 MH3B1 binding both soluble and cell surface expressed HER2/*neu*. hDM-C6 MH3B1 is relatively stable in the presence of serum at 37°C. Comparison of the structures of hDM and the wild type enzyme as well as analysis of potential MHCII binding peptides generated as a result of fusion of two proteins and the Glu201Gln:Asn243Asp mutations suggest that hDM-αH-C6.5 MH3B1 should have minimal immunogenicity in humans. Therefore, the hDM-C6 MH3B1-F-dAdo combination addresses many of the current limitations of ADEPT and provides an excellent candidate for treatment of HER2/*neu *expressing tumors with minimum systemic toxicity or immunogenicity.

## Methods

### Materials

Guanosine and F-Ade were purchased from Sigma-Aldrich (St. Louis, MO), and F-dAdo was purchased from Berry & Associates (Dexter, MI). CT26 was purchased from ATCC **(**Manassas, VA). Construction and characterization of CT26HER2/*neu *is described previously [8]. MCF7-HER2 [9] was a gift from Dr. Dennis Slamon (University of California, Los-Angeles). Cells were cultured in Iscove's Modified Dulbecco's Medium (IMDM; GIBCO, Carlsbad, CA) containing 5% calf-serum (GIBCO) for CT26 and CT26HER2/neu and IMDM containing 10% fetal bovine serum (GIBCO), 1% non-essential amino acids (GIBCO) and 1% sodium pyruvate (GIBCO) for MCF-7HER2 cells. The expression vector for production of ECD^HER2 ^was a gift from Dr. James Marks (University of California, San-Francisco).

### Plasmid construction and protein purification

Cloning of hPNP and hDM with αH linker at its C-terminus was described previously [5]. To construct hPNP-αH-C6.5 MH3B1 or hDM-αH-C6.5 MH3B1 genes, first PNP-αH was amplified using 5'**gcggccgc**gataccaccgatatccaccatggagacagacacactcctgctatgggtactgctgctctgggttccaggttccactggagacgagaatggatac acctatgaagattataagaac3' and 5'taaagaggccgcagccaaagcgcaggtgcagctggtgcagtctgg3' as forward and reverse primers respectively. The forward primer contains a NotI site, Kozak sequence and signal peptide, and the beginning of the PNP gene. The reverse primer encodes the αH linker and the beginning of C6.5 MH3B1. The sequence for the signal peptide is gatatccaccatggagacagacacactcctgctatgggtactgctgctctgggttccaggttccactggagac. The amino acid sequence for the αH linker is AEAAAKEAAAKA. The C6 MH3-B1 gene was PCR amplified with the forward primer complementary to the reverse primer used for PNP amplification encoding for αH linker, and the beginning of the C6.5 MH3B1 sequence. 5'ggagggaccaaggtcaccgtcctaggtcgttaataa**tctaga**3', which encodes the C-terminus of scFv and an XbaI site, was used for the reverse primer. The PCR product of each gene was purified, annealed and used as template for the final PCR amplification using PNP forward primer containing a NotI site and C6.5 MH3B1 reverse primer containing a XbaI site. The PCR product was cloned into the TOPO-Blunt vector (Invitrogen, Carlsbad, CA) and the sequence confirmed. The final product encoding the Kozak sequence, signal sequence, hPNP or hDM followed by an αH linker and C6.5 MH3B1 was cloned into the Novagen vector pcDNA3.1 (+) using NotI and XbaI sites.

### Expression, purification and SDS PAGE

The pcDNA3.1 (+) vector containing the insert was transiently transfected into 293T cells using CalPhos Mammalian Transfection Kit (Clonetech Laboratories, Inc. Mountain View, CA) according to manufacturer's recommendation. Culture supernatant was collected three and six days post transfection and passed through an affinity column that consisted of ECD^HER2 ^conjugated to CNBr activated Sepharose beads according to manufacturer's recommendation. The affinity column was washed with 30 column volumes of PBS, and 3 column volumes of acetic acid pH 4.5. The bound protein was eluted with 0.1 M glycine pH 2.5 and immediately neutralized with Tris/HCl pH 8.0. Protein concentration was determined by absorbance at 280 nm using E^0.1% ^= 1.6 with molecular mass of 60,392 Da, and the protein purity was assessed using Coomassie blue-stained SDS polyacrylamide gel. Expression and purification of ECD^HER2 ^is described previously [8].

### Size exclusion analysis of hDM-αH-C6.5 MH3B1

To determine whether hDM-αH-C6.5 MH3B1 exists as monomers and/or as polymers, 100 μg of purified protein was analyzed by gel filtration on a Superose 6 HR 10/30 column (GE Healthcare, Anaheim, CA) by HPLC in PBS at 0.2 ml/min. BIORAD gel filtration standards (catalog # 151-1901; Hercules, CA) composed of Thyroglobulin (670,000 Da), γ-globulin (158,000 Da), Ovalbumin (44,000 Da), Myoglobin (17,000 Da), and Vitamin B12 (1,350 Da) were used as molecular weight standards.

### Enzyme activity and kinetic parameters of PNP fusionproteins

The method for determining the enzymatic activity of hPNP or any of its mutant constructs was previously described in detail [5]. Briefly, enzymatic cleavage of F-dAdo to F-Ade by PNP was followed by a decrease in absorbance at 260 nm and a concurrent increase in absorbance at 280 nm with a molar extinction coefficient of 16,300 M^-1^cm^-1 ^at 260 nm and 1,300 M^-1^cm^-1 ^at 280 nm. Phosphorolysis of guanosine to guanine was followed by the decrease in absorbance at 257 nm using a molar extinction coefficient of 13,700 M^-1^cm^-1 ^for guanosine.

### Association of hDM-αH-C6.5 MH3B1 with HER2/neu expressingcells

CT26, CT26-HER2/*neu*, and MCF-7HER2 cells were seeded at 5 × 10^3 ^cells in 50 μl per well in a 96-well microtiter plate. CT26 and CT26-HER2/*neu *were grown in the presence of Iscove's Modified Dulbecco's Medium (GIBCO; Carlsbad, CA) containing 5% calf-serum (GIBCO). MCF-7HER2 cells were grown in the presence of ISCOVE's Modified Dulbecco's Medium containing 10% Fetal Bovine Serum (GIBCO), 1% Non-essential amino acids (GIBCO), and 1% Sodium Pyruvate (GIBCO). The next day 50 μl of increasing concentrations of hDM-αH-C6.5 MH3B1 were added in triplicate to cells and incubated for 45 minutes at room temperature. The unbound proteins were carefully pipetted out, and each well was gently washed twice with 200 μl of cold medium. This was followed by addition of 100 μl of growth medium containing F-dAdo at a final concentration of 1.5 μM for CT26 or CT26-HER2/neu cells or 6 μM for MCF-7HER2 cells [5]. After 72 hours incubation at 37°C, inhibition of cell growth was determined by an MTS assay according to manufacturer's recommendation. When the fusion proteins were directly added, cells were seeded as described above. Then 40 μl of fusion protein at different dilutions and 10 μl of F-dAdo stock (1.5 μM for CT26 or CT26HER2/neu cells and 6 μM for MCF-7HER2 cells) were added to cells and incubated for 72 hours at which time the degree of cell proliferation was determined by MTS assay. To examine the bystander effect of the fusion protein, mixtures of CT26 and CT26HER2/neu cells were seeded overnight at 5 × 10^3 ^cells per well at different ratios, and the assay was completed as described above with hDM-αH-C6.5 MH3B1 and F-dAdo at final concentrations of 0.1 μM and 1.5 μM, respectively.

### Cytotoxicity of F-Ade to cells with different growth rates

MCF7-HER2 cells were seeded overnight at a density of 5 × 10^3 ^in the presence of 10% fetal bovine serum. The following day, cells were washed carefully, the medium replaced with serum at different levels to influence growth rate, and cells grown for an additional 72 hours in the presence or absence of 6 μM F-Ade. The level of cell viability or the number of cells were determined by MTS assay, or by visually counting them.

### Stability of hDM-αH-C6.5 MH3B1 at 37°C in serum

To evaluate the stability of hDM-αH-C6.5 MH3B1, hDM-αH-C6.5 MH3B1 at a concentration of 0.001 μM was incubated in the presence of fetal bovine serum at 37°C for up to 23 hours. Samples were removed at different times and stored at 4°C. After the last sample was removed, each was added to overnight seeded MCF-7HER2 cells (5 × 10^3^/well) in the presence of 6 μM F-dAdo, and the activity of hDM-αH-C6.5 MH3B1 was determined by its ability to convert F-dAdo to F-Ade and inhibit cell proliferation as assessed by MTS assay 72 hours after addition of fusion protein and prodrug to cells.

### SPR analysis of interaction of ECD^HER2 ^with hDM-αH-C6.5 MH3B1

inding of hDM-αH-C6.5 MH3B1 to ECD^HER2 ^was evaluated using surface plasmon resonance (SPR) on a BIAcore T-100. To determine the affinity of the monomeric interaction of hDM-αH-C6.5 MH3B1 with ECD^HER2^, 533 resonance units (RU) of trimeric hDM-αH-C6.5 MH3B1 were immobilized on the surface of a CM5 sensor chip following the standard amine coupling procedure according to the manufacturer's suggestion. The remaining active groups were blocked by ethanolamine. A control surface was generated by following the same procedure, but without addition of protein. ECD^HER2 ^at concentrations ranging from 10 to 100 nM in PBS was flowed over the surface at 30 μl/min for 750 second. This was followed by a 45 minute dissociation phase at the same flow rate. The surface was regenerated by two injections of glycine at pH 9.5 for 20 seconds. As control, PBS alone or a mixture of 100 nM ECD^HER2 ^and 1 μM hDM-αH-C6.5 MH3B1 incubated at 25°C for 30 minutes was injected on the surface. Binding of ECD^HER2 ^to immobilized hDM-αH-C6.5 MH3B1 was monitored in real time by following the association and dissociation phases on the experimental surface with control surface subtracted. Binding parameters were determined using the 1:1 binding model by BIAevalution 3.0 software.

### Flow cytometry analysis of hDM-αH-C6.5 MH3B1 binding to HER2/neu expressing cells

CT26, CT26HER2/*neu *or MCF-7HER2 cells (5 × 10^5 ^cells/sample) were incubated with either biotinylated or Alexa-fluor labeled hDM-αH-C6.5 MH3B1 for 30 minutes on ice and then washed twice with FACS buffer [PBS pH 6.8 with 1% calf-serum]. If biotinylated hDM-αH-C6.5 MH3B1 was used, cells were then stained for thirty minutes with PE-labeled streptavidin at final concentration of 0.3 μg/ml (BD Bioscience; Franklin Lakes, NJ), and washed twice. Fluorescence was measured on a cytofluorometer (FACSCalibur; BD Bioscience) and the mean fluorescence was analyzed using the Flowjo software (Treestar, Ashland, OR). Biotin (catalog number: 21336; PIERCE; Rockford, IL) or Alexa-fluor (catalog number: A10235; Invitrogen) conjugation of hDM-αH-C6.5 MH3B1 was carried out according to the manufacturer's recommendation.

## Results

### Construction and purification of hDM-αH-C6.5 MH3B1

**Figure 1:**
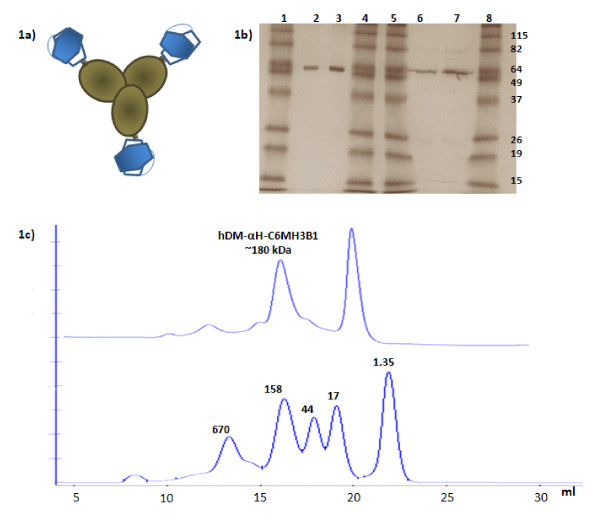
**Schematic presentation and purity ofhDM-αH-C6 MH3B1**. **(A)**, Schematic diagram of hDM-αH-C6 MH3B1. Each monomer of hDM is shown as filled oval. The αH linker, fused to the C-terminus of hDM is shown in black and C6 MH3B1 is shown as hexagons. **(B)**, SDS-PAGE analysis under non-reducing and reducing conditions of purified hDM-αH-C6.5 MH3B1 visualized by Coomassie Blue staining. *Lanes *1, 4, 5, and 8, MW markers in kDa (Invitrogen); *lanes *2 & 3, hDM-αH-C6.5 MH3B1 at 1 and 2 μg, respectively, not reduced; lane 6 & 7, hDM-αH-C6.5 MH3B1 at 1 and 2 μg, respectively, reduced. **(C)**, Size exclusion chromatography of hDM-αH-C6.5 MH3B1 under non-reducing condition using a Sepharose-6 column. For comparison, molecular weight standards were analyzed under identical conditions.

We have previously shown that hPNP with the two mutations Glu201Gln:Asn243Asp, unlike wild-type hPNP, converts a relatively non-toxic prodrug, F-dAdo to the cytotoxic drug F-Ade [5]. With the goal of being able to target hDM to the tumor site, we fused it at its C-terminus to a human anti-HER2/*neu *single chain Fv (C6.5 MH3-B1) [7] through a rigid α-helical linker [10,11] (Fig. 1A). C6.5 MH3B1 has been reported to bind to HER2/*neu *with high affinity and specificity [7]. The available crystal structure of hPNP [12-14] suggested that fusing C6.5 MH3B1 to the C-terminus of the enzyme would have minimal affect on its enzymatic activity, since the C-terminus is distal from the enzyme active site. The rigid α-helical linker [11,12], instead of a flexible GlySer linker was used to restrict the flexibility of the fusion protein. The plasmid encoding the hDM-αH-C6 MH3B1 was transiently expressed in 293T cells, the supernatant harvested and the protein purified by passage through an affinity column composed of ECD^HER2 ^conjugated to Sepharose beads. The eluted protein was 99% pure as judged by Coomasie blue staining with 300 μg of protein obtained from 150 ml of culture supernatant (Fig. 1B). Analysis of the protein by size exclusion chromatography indicated that the fusion protein mainly existed as a 180 kDa homotrimer (Fig. 1C) of 60 kDa subunits.

### hDM-αH-C6.5 MH3B1 unlike hPNP-αH-C6.5 MH3B1 converts the non-toxic prodrug F-dAdo to the cytotoxic drug, F-Ade

**Figure 2:**
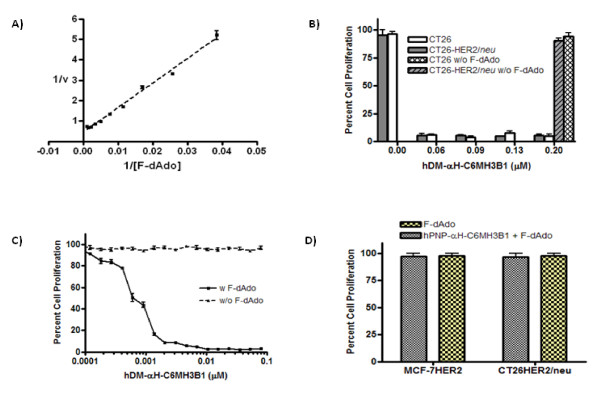
**Enzymatic activity of hDM-αH-C6.5 MH3B1**. **(A)**, Lineweaver-Burk plot of enzyme activity of hDM-αH-C6.5 MH3B1 with F-dAdo as substrate. Conversion of F-dAdo to F-Ade was followed spectrophotometrically in real time by the increase in absorbance at 280 nm. Concentration of F-dAdo is in μM and v is based on mili-units of absorbance/min. **(B)**, Proliferation of CT26 and CT26HER2/*neu *cells and **(C)**, MCF-7HER2 cells in the presence or absence of F-dAdo or hDM-αH-C6.5 MH3B1 was determined in 72 hours by MTS. **(D)**, 0.2 μM of hPNP-αH-C6.5 MH3B1 was incubated with CT26HER2/*neu *or MCF-7 cells in the presence of 1.5 or 6 μM of F-dAdo respectively for 72 hours and cellular proliferation determined by MTS assay. Error bars for each graph represent standard deviation within each set of values.

The activity of hDM-αH-C6 MH3B1 was examined in a spectrophotometeric assay in which conversion of F-dAdo to F-Ade was followed by a decrease in absorbance at 260 nm and a concurrent increase in absorbance at 280 nm. The fusion protein had a *K*_*M *_of 264 μM and a *k*_*cat *_of 0.155 s^-1 ^with an overall efficiency of 586 M^-1^s^-1 ^(Fig. 2A, Table 1). When compared to the enzymatic activity of hDM fused to a short **a**nti-**H**ER2/***n****eu ***p**eptide called AHNP [5,15], hDM-αH-C6.5 MH3B1 showed a two-fold reduction in *K*_*M *_with a two-fold increase in *k*_*cat*_, with the overall efficiency of the enzyme remaining unchanged with respect to F-dAdo. Unlike the wild-type PNP, enzymatic activity of hDM-αH-C6.5 MH3B1 with respect to guanosine was weak (data not shown). A cell based assay confirmed that the fusion protein converts F-dAdo to a cytotoxic agent. First, a concentration of F-dAdo was determined that was not toxic to cells, but if converted to F-Ade, would inhibit cellular proliferation; this concentration was 1.5 μM for CT26 or CT26HER2/*neu *and 6 μM for MCF-7HER2 cells. CT26 or CT26Her2/*neu *cells grew normally when either 1.5 μM of F-dAdo or 0.2 μM of hDM-αH-C6.5 MH3B1 was added (Fig. 2B), but when added together, F-dAdo was converted to F-Ade by hDM and cell proliferation was inhibited (Fig. 2B). In a similar experiment using MCF7-HER2 cells, addition of 6 μM F-dAdo or 0.1 μM of hDM-αH-C6.5 MH3B1 did not affect cell proliferation (Fig. 2C); however, addition of hDM-αH-C6.5 MH3B1 in the presence of 6 μM F-dAdo inhibited cell proliferation in a dose dependent manner with half-maximum inhibition of proliferation at 0.6 nM, and complete inhibition of cell proliferation at 2 nM (Fig. 2C). Since no toxicity was seen with 6 μM of F-dAdo or 0.1 μM of hDM-αH-C6.5 MH3B1 (Fig. 2C), the observed cytotoxicity must be the result of the conversion of F-dAdo to F-Ade through the enzymatic activity of hDM. In summary, F-dAdo is toxic to cells only when cleaved to the cytotoxic drug, F-Ade by hDM-αH-C6.5 MH3B1. Significantly, F-Ade inhibits proliferation of a variety of cell types including the murine colon carcinoma CT26 or CT26HER2/*neu *and the human breast cancer line MCF7-HER2, as well as melanoma tumor cell line, B16 and murine B-cell tumor cells, 38C13 (data not shown).

**Table 1:** Kinetic constants of fusion protein for F-dAdo as substrate.

	*K*_*M*_ (μM)	*K*_*cat*_ (s^-1^)	*k*_*cat*_/*K*_*M*_ (M^-1^s^-1^)
hPNP-αH-C6 MH3B1	nd	nd	Nd

hDM-αH-C6 MH3B1	264 ± 22	0.155 ± 0.017	568.2

nd: no activity detected

Addition of hPNP-αH-C6.5 MH3B1 and F-dAdo to either MCF7-HER2 or CT26-HER2/*neu *cells did not result in cytotoxicity (Fig. 2D), consistent with the fact that the wild type enzyme cannot use F-dAdo as substrate (Table 1). However, hPNP-αH-C6.5 MH3B1 is able to cleave its natural substrate, guanosine, although with a *K*_*M *_of 59 μM, a *kcat *of 60 s^-1 ^and an overall efficiency of 1 × 10^6 ^M^-1^s^-1 ^(Table 2) that is 3 to 7-fold less than the reported values for the free enzyme [5,6].

**Table 2:** Kinetic constants of hPNP-αH-C6 MH3B1 for guanosine as substrate.

	*K*_*M*_ (μM)	*K*_*cat*_ (s^-1^)	*k*_*cat*_/*K*_*M*_ (M^-1^s^-1^)
hPNP-αH-C6 MH3B1	59 ± 10	60 ± 13	1.02 × 10^4^

### Stability of hDM-αH-C6.5 MH3B1 at 37°C in the presence of serum

**Figure 3:**
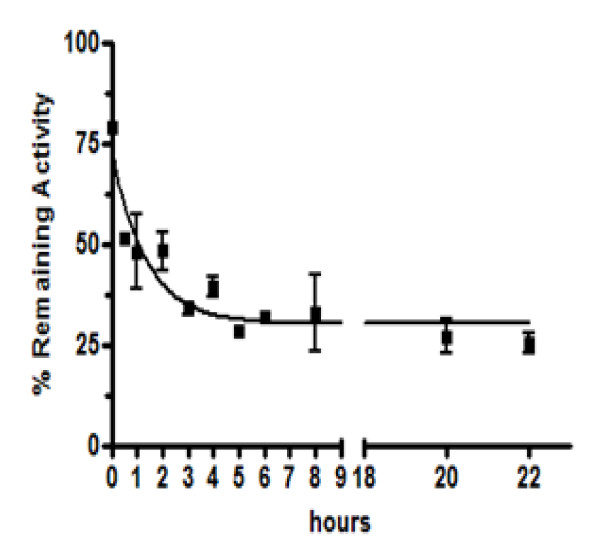
**Stability of hDM-αH-C6.5 MH3B1 at 37°C in the presence of serum**. hDM-αH-C6.5 MH3B1 was either stored in PBS at 4°C or incubated for various times at 37°C in the presence of serum. After incubation at 37°C, fusion protein was stored at 4°C until the experiment was completed (~23 hours). hDM-αH-C6.5 MH3B1 was then added to MCF-7HER2 cells and its enzymatic stability was evaluated by its ability to convert F-dAdo to F-Ade resulting in inhibition of cellular proliferation. Data are shown as percent activity remaining of 0.001 μM of hDM-αH-C6.5 MH3B1 incubated in serum at 37°C for various times relative to the activity of 0.001 μM of hDM-αH-C6.5 MH3B1 in PBS at 4°C. The error bars represent standard deviation within each set of values.

The stability of hDM-αH-C6.5 MH3B1 in serum at 37°C was evaluated by its ability to cleave F-dAdo to F-Ade. It was expected that different concentrations of F-Ade would be produced depending on the activity of the added enzyme. It had previously been determined that at a concentration of 0.001 μM, the activity of hDM-αH-C6.5 MH3B1 is limiting (Fig. 2C), and hence any partial or complete loss in its activity would be measurable. Therefore, 0.001 μM of hDM-αH-C6.5 MH3B1 was either stored in PBS at 4°C or incubated with fetal bovine serum at 37°C for various times, followed by immediate transfer to 4°C until completion of the assay (~23 hours). Different aliquots of the fusion protein were added to MCF-7HER2 cells in the presence of 6 μM F-dAdo, and following incubation for 72 hours at 37°C, cell proliferation was determined by the MTS assay. As shown in Figure 3, incubation of the fusion protein overnight at 4°C in the presence of serum resulted in loss of activity compared to the enzyme that was incubated in PBS. When the fusion protein was incubated in serum at 37°C, a time dependent loss in activity was observed. However, even after 23 hours at 37°C in the presence of serum, some enzyme activity remained (Fig. 3). Consistent with these findings, when a 10-fold higher concentration (0.01 μM) of fusion protein was used, after 23 hours at 37°C sufficient enzyme activity remained to cleave F-dAdo and result in complete inhibition of cell proliferation (data not shown).

### hDM-αH-C6.5 MH3B1 binds to HER2/neu with high affinity and specificity

**Figure 4:**
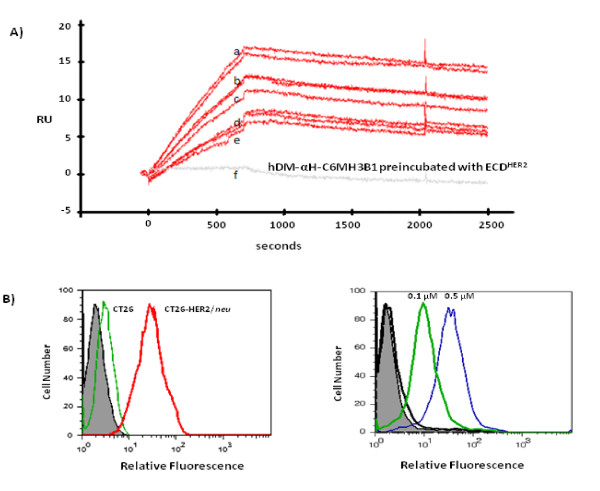
**Binding of hDM-αH-C6.5 MH3B1 to ECD^HER2^**. **(A)**, Interaction of ECD^HER2 ^with hDM-αH-C6.5 MH3B1 immobilized on the surface of a SPR chip. ECD^HER2 ^at concentrations of a) 100 nM in duplicate b) 50 nM in duplicate c) 40 nM d) 20 nM in duplicate or e) 10 nM was flowed across the sensor chip at 30 μl/min for 750 second. f) Binding of 100 nM ECD^HER2 ^to immobilized hDM-αH-C6.5 MH3B1 after incubation with 1 μM hDM-αH-C6.5 MH3B1. **(B)**, Binding of biotinylated hDM-αH-C6.5 MH3B1 to ECD^HER2 ^expressed on the cell surface. Bound protein was detected using Streptavidin-PE. Left panel shows binding of 0.5 μg of biotinylated hDM-αH-C6.5 MH3B1 to CT26HER2/*neu *and not to the parental cells that lack HER2/*neu *expression. Right panel shows binding of 0.1 μg (heavy green), or 0.5 μg of biotinylated hDM-αH-C6.5 MH3B1 (thin blue) or Streptavidin-PE (heavy black) to MCF-7HER2 cells. Filled are unstained cells.

The specific interaction of hDM-αH-C6.5 MH3B1 with ECD^HER2 ^was demonstrated using three different approaches. First, binding of hDM-αH-C6.5 MH3B1 to ECD^HER2 ^conjugated to Sepharose beads was used to purify the fusion protein. Treatment with glycine pH 2.5 was required to elute the bound protein, consistent with a strong interaction between hDM-αH-C6.5 MH3B1 and ECD^HER2^. In a second approach, the interaction was evaluated using surface plasmon resonance. hDM-αH-C6.5 MH3B1 and ECD^HER2 ^exist as a trimer (Fig. 1) and a monomer respectively. To make the analysis of the binding more straightforward, trimeric hDM-αH-C6.5 MH3B1 was immobilized on the sensor chip, so that the measured binding should represent the interaction of a single binding site of hDM-αH-C6.5 MH3B1 with monomeric ECD^HER2^. Different concentrations of ECD^HER2 ^were flowed for 750 seconds over immobilized hDM-αH-C6.5 MH3B1 at 30 μl/min (Fig. 4A), and binding was observed as an increase in RUs. From these data, the binding affinity of hDM-αH-C6.5 MH3B1 to ECD^HER2 ^was calculated using a 1:1 binding model to be 3.4 × 10^-10 ^M, with a k_on _of 1.7 × 10^4 ^M^-1^s^-1 ^and a K_off _of 5.8 × 10^-6 ^s^-1^, values similar to what had been observed with single chain C6.5 MH3B1 [7]. Incubation of ECD^HER2 ^with hDM-αH-C6.5 MH3B1 prior to the injection prevented the binding of ECD^HER2 ^to immobilized hDM-αH-C6.5 MH3B1 (Fig. 4A, a-f). In a third approach, the interaction of hDM-αH-C6.5 MH3B1 with ECD^HER2 ^expressed on the cell surface was analyzed by flow-cytometry. Biotinylated hDM-αH-C6.5 MH3B1 bound specifically to CT26HER2/*neu *cells and not the parental CT26 cells that lack expression of HER2/*neu *(Fig. 4B). Biotinylated hDM-αH-C6.5 MH3B1 also bound to MCF-7HER2 cells (Fig. 4B). In summary, hDM-αH-C6.5 MH3B1 interacts specifically and with high affinity with both soluble and cell-expressed ECD^HER2^.

### hDM in hDM-αH-C6.5 MH3B1 can target cytotoxic activity to HER2/neu expressing cells

**Figure 5:**
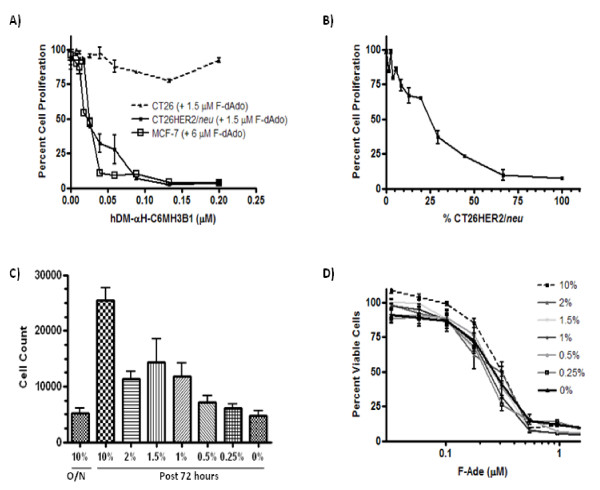
**hDM-αH-C6.5 MH3B1 specifically associates with HER2/*neu *expressing cells and causes cytotoxicty in the presence of F-dAdo irrespective of expression of tumor antigen or cell growth rate**. **(A)**, hDM-αH-C6.5 MH3B1 associates with HER2/*neu *expressing cells resulting in concentration dependent cytotoxicity upon addition of 1.5 or 6 μM F-dAdo to CT26HER2/*neu *or MCF-7HER2 cells respectively. Different concentrations of hDM-αH-C6.5 MH3B1 were incubated with cells, unbound enzyme washed away, F-dAdo added and 72 hours later cellular proliferation was determined by MTS assay. **(B)**, CT26HER2/*neu *and CT26 cells were seeded at different ratios and grown overnight. hDM-αH-C6.5 MH3B1 was incubated with cells for 45 minutes, and washed away. Cells were then grown in the presence of 1.5 μM F-dAdo for 72 hours and cell proliferation determined by MTS assay. **(C)**, MCF-7HER2 cells were grown overnight (O/N) in the presence of 10% serum, washed and growth continued for 72 hours in the presence of varying amounts of serum. The column labeled overnight (O/N) represents the number of cells prior to switching to different amounts of serum. The number of cells was determined by visually counting the cells. **(D)**, Viability of MCF-7HER2 cells in the presence of different amounts of fetal bovine serum and 1.5 μM F-Ade was determined after 72 hours of incubation by MTS assay. Error bars for each graph represent standard deviation within each set of values.

To determine if hDM-αH-C6.5 MH3B1 activity can be specifically targeted to HER2/*neu *expressing cells, fusion protein was incubated at room temperature for 45 minutes with CT26HER2/*neu*, the parental CT26 cells that lack the expression of HER2/*neu *or MCF-7HER2. The unbound protein was washed away, 1.5 μM or 6 μM of F-dAdo added, and after 72 hours the amount of cell proliferation was determined by MTS. hDM-αH-C6.5 MH3B1 was found to remain bound to HER2/*neu *expressing cells, causing a dose dependent inhibition of cell proliferation in the presence of F-dAdo as a consequence of its conversion to F-Ade. No cytotoxicity was seen with CT26 cells that did not express HER2/*neu *(Fig. 5A). For CT26HER2/*neu *and MCF-7HER2 cells the IC_50 _for hDM-αH-C6.5 MH3B1 was 0.0196 μM and 0.0254 μM, respectively. In summary, enzymatic activity of hDM-αH-C6.5 MH3B1 remains associated with HER2/*neu *expressing cells and causes cleavage of F-dAdo to F-Ade resulting in dose dependent inhibition of cell proliferation.

### Conversion of F-dAdo to F-Ade by cell bound hDM-αH-C6.5 MH3B1 results in bystander activity

For ADEPT to be effective, the cytotoxic drug generated by the activity of the cell associated enzyme should be cytotoxic to the neighboring cells that may lack the expression of the tumor associated antigen. To investigate the bystander effect of F-Ade generated by the enzymatic activity of hDM-αH-C6.5 MH3B1, different ratios of CT26HER2/*neu *and CT26 cells were mixed and seeded. The next day, cells were incubated with 0.1 μM of hDM-αH-C6.5 MH3B1 for 45 minutes, washed twice, and after 72 hours the level of inhibition of cell proliferation caused by F-Ade that was generated by the enzymatic activity of bound hDM-αH-C6.5 MH3B1 was determined by MTS assay. Complete inhibition of cell proliferation was achieved when up to 35% of the seeded cells were comprised of CT26 (Fig. 5B). When 75% of the cells were CT26, 50% inhibition of cell growth was observed (Fig. 5B). This result indicates that the F-Ade generated by the enzymatic activity of hDM-αH-C6.5 MH3B1 bound to CT26HER2/*neu *is not only toxic to HER2/*neu *expressing cells, but also to the neighboring cells that lack the expression of tumor antigen.

### F-Ade is toxic to rapidly, slowly and non-dividing cells

Since it has been shown that the non-dividing stromal cells play a critical role in providing support for tumor growth, and since tumors are composed of cells growing at different rates, we examined the cytotoxic affect of F-Ade on slowly-dividing or non-dividing cells. MCF-7HER2 cells were grown overnight in growth medium that contained 10% fetal bovine serum. The next day, cells were washed and incubated for 72 hours in medium that contained varying amounts of serum. MCF-7HER2 cells divided even with serum levels as low as 0.25% and ceased to divide, but remained viable only when no serum was present (Fig. 5C). In the presence of different concentrations of F-Ade, similar cytotoxicity was observed irrespective of the rate of cell growth (Fig. 5D). This indicates that F-Ade is toxic to the rapidly or slowly growing tumor cells as well as to the non-dividing neighboring cells that may sustain tumor growth.

### Novel MHCII binding peptides present in hDM-αH-C6 MH3B1

B cells are activated to develop into antibody producing plasma cells when their B cell receptor interacts with non-self epitopes on soluble proteins and when they receive a signal from T_H _cells. It seems likely that hDM-αH-C6 MH3B1 will exhibit minimal reactivity with the B cell receptor because the two introduced mutations are buried within the purine binding pocket of hDM and the structure of hDM is extremely similar to the structure of wild type enzyme [13]. A recently developed evaluation tool which identifies peptides that might bind to MHCII molecules [16] was used to identify potential MHCII binding peptides present in hDM-αH-C6 MH3B1. After the peptides common between hDM and hPNP were eliminated, 10 and 1 new possible binders that were generated as a result of Glu201Gln and Asn243Asp mutations respectively were identified. Although, hDM and C6 MH3B1 are both human derived proteins, novel MHCII binding peptides may result from their fusion. To address this possibility, we also evaluated a 40 amino acid long peptide that included 14 amino acids from the C-terminus of hDM, the complete sequence of the α-helical linker and a 14 amino acids stretch of the N-terminus of C6 MH3B1 for possible MHCII binding peptides [16]. Only 6 potential MHCII binding peptides for all human MHCII alleles were identified suggesting that minimal immunogenicity should result from the fusion of hDM to C6 MH3B1. Therefore, the probability of hDM-αH-C6 MH3B1 inducing a robust immune response in human should be minimal.

## Discussion

In order to develop a clinically relevant non-immunogenic therapeutic approach to ADEPT, we fused a mutant human enzyme to a human scFv specific for the HER2/*neu *tumor antigen. ADEPT requires both an active enzyme and the ability to target that enzyme to the tumor. Here we show that fusion of the mutant human PNP to the anti-HER2/*neu *scFv via an α-helical linker (hDM-αH-C6.5 MH3B1) results in an active protein that can be targeted to tumor cells, where it can cleave a relatively non-toxic prodrug to a cytotoxic drug, resulting in the inhibition of tumor cell proliferation.

Previously it was shown that fusion of a 1.5 kDa short **a**nti-**H**ER2/***n****eu ***p**eptide (AHNP) to the C-terminus of hDM did not result in loss of enzyme activity [5]. We have now extended these studies to show that replacement of AHNP with the much larger (~50 kDa) scFv also did not significantly affect the activity of hDM (Table 1). In this fusion protein, a rigid α-helical linker was used to join the two domains. The spacing provided by the inflexible linker may minimize steric hinderace that could adversely influence the activity of either hDM or C6.5 MH3B1. Moreover, the C-terminus of the enzyme is extended away from the enzyme active site; therefore, fusion of a targeting component to the C-terminus of hDM should have a minimal affect on substrate binding and catalysis. Since hDM remains active after fusion to C6.5 MH3B1, it is reasonable to expect that following fusion of other scFvs with different specificities to hDM, the enzyme will remain active and capable of being targeted to other tumors. Therefore, the use of hDM is not restricted to HER/*neu *expressing tumors, but should be useful for ADEPT therapy of a wide variety of cancers.

Fusion of hDM to the single chain C6.5 MH3B1 resulted in specific association of the enzyme activity with the HER2/*neu *expressing cells (Fig. 5A). C6.5 MH3B1 was chosen to target hDM to the tumor site based on its specificity, high affinity and slow dissociation rate from the HER2/*neu *tumor antigen [7]. SPR analysis of the binding affinity of hDM-αH-C6.5 MH3B1 to ECD^HER2 ^showed a strong binding affinity of 3.4 × 10^-10 ^M, approximately three fold less strong than that of the single chain C6.5 MH3B1 [7]. The trimeric structure of hDM-αH-C6.5 MH3B1 should further increase its binding to cell associated HER2/*neu*. The high affinity should ensure that hDM-αH-C6.5 MH3B1 effectively targets the tumor and persists at the tumor site long enough to allow the systemically administered F-dAdo to reach the tumor and be cleaved to F-Ade [5,7,17,18]. It has been suggested that high affinity scFvs would mainly be retained in the perivascular regions of the tumor where the first tumor antigen is encountered [19], preventing tumor penetration. While this might weaken the clinical applicability of some therapeutic scFvs, it should not be an issue for ADEPT. In fact, retention of hDM-αH-C6.5 MH3B1 on the cell surface in the tumor microenvironment for an extended period of time should make the enzyme readily accessible for cleaving the prodrug to a cytotoxic drug.

Properties of hDM-αH-C6.5 MH3B1, such as thermal stability and resistance to proteolysis contribute to its effectiveness *in vitro *and *in vivo*. When hDM-αH-C6.5 MH3B1 was incubated with serum at 37°C only 50% of enzyme activity was recovered after 30 minutes (Fig. 3). Longer incubation resulted in a further rapid loss of activity so that after 3 hours only about 30% of the activity remained. However, further incubations for 21 hours resulted in little further decrease in activity (Fig. 3). Incubation with serum over night at 4°C resulted in a 20% loss of activity (Fig. 3). The observed loss of enzyme activity in the presence of serum is most probably due to degradation of the protein by the serum proteases and the small additional decrease in enzyme activity following 3 hours of incubation may indicate that the serum proteases themselves become inactivated upon incubation and lose activity by 3 hours. Alternatively, there may exist different conformers of hDM-αH-C6.5 MH3B1 that exhibit different stabilities in serum.

The use of hDM with F-dAdo constitutes a novel and specific enzyme-prodrug combination. Addition of hDM-αH-C6.5 MH3B1 alone, F-dAdo alone or hPNP-αH-C6.5 MH3B1 with F-dAdo, did not affect cell proliferation. This is particularly important since hPNP is a ubiquitous enzyme present at micromolar concentrations in blood cells [12]. Therefore, lack of activity of hPNP-αH-C6.5 MH3B1 with F-dAdo should reduce toxicity concerns in vivo. However, when hDM-αH-C6.5 MH3B1 was added to cells in the presence of F-dAdo, the cytotoxic F-Ade generated due to enzymatic activity of hDM-αH-C6.5 MH3B1 resulted in a dose-dependent inhibition of cell proliferation (Fig. 2C). Our *in vitro *studies have shown that F-dAdo conversion to F-Ade occurs by hDM that is targeted to tumor cells through specific interaction of C6.5 MH3B1 with cell expressed HER2/*neu*. However, the generated F-Ade is toxic to all tumor cells regardless of their expression of tumor antigen. When 65% of cells express HER2/*neu*, enzymatic activity of hDM-αH-C6.5 MH3B1 that is bound on their cell surface, resulted in generation of sufficient F-Ade to inhibit proliferation of all the tumor cells, regardless of their expression of HER2/*neu *(Fig. 5B). While the mechanism of F-Ade passage from cell to cell is not exactly known, it has been shown to be independent of gap junctions and does not require cell-cell contact [20,21]. In addition to being able to kill the rapidly dividing tumor cells, F-Ade can also cross the cell membrane of the slowly-dividing or even non-dividing neighboring cells and cause cytotoxicity (Fig. 5D). This is especially important since tumors are heterogeneous and are composed of cells with different growth rates. Moreover, neighboring stromal cells that do not divide play an important role in supporting tumor growth. Therefore, F-Ade that inhibits DNA, RNA as well as protein synthesis [22], can effectively cause growth arrest in all cell types that contribute to tumor survival, while exerting minimal toxicity to the distal healthy cells due to its expected short half-life of only 5 hours *in vivo *[22].

An important consideration is whether hDM-αH-C6 MH3B1 will induce an immune response in humans. Generation of antibodies against the bacterial enzyme and the murine targeting component in patients receiving ADEPT has to date prevented further treatment [2,23]. Antibodies are produced against foreign substances by activated differentiated B cells that have bound to non-self epitopes and received signals from an activated T_H _cell. In hDM, the two introduced mutations, E201Q:N243D are buried within the enzyme; hence, they are not directly accessible to bind the B cell receptor. Moreover, we have shown that the overall structure of hDM with F-dAdo is very similar to that of hPNP complexed with its natural substrate, guanosine. Although, the presence of neo-epitopes cannot be dismissed, it is anticipated that the mutant enzyme should have minimal reactivity with the B cell receptor. Additionally, fusion is achieved by using a rigid αH linker, whose rigidity should make the whole molecule less flexible and therefore less immunogenic [24,25].

In contrast to the B cell receptor, the T cell receptor recognizes non-self epitopes by recognizing protein-derived peptide fragments bound to MHC molecules. Therefore, T-cells can react to foreign epitopes that are buried within a protein. To be recognized by T cells, the peptides must first bind to MHC molecules expressed on the surface of antigen presenting cells. However, binding of a peptide to MHCII does not necessarily result in T_H _cell activation, and only 9.4% of the predicted binders have been found to actually activate T cells [16]. Given that only 11 new possible binders are predicted to result from the introduced mutations, the likelihood of hDM activating T_H _cells is small. Therefore, we propose that hDM should be far less immunogenic than the currently used bacterial enzymes.

## Conclusion

In this study, we have demonstrated the feasibility of ADEPT in which both the enzyme and the targeting moiety are of human origin. Our study has shown that hDM, a version of human PNP with only two amino acid substitutions, can be fused to a targeting component comprised of a human-derived scFv without loss of activity. Moreover, we have shown that the drug generated by the enzymatic activity of hDM causes tumor cell death regardless of their expression of tumor associated antigen or growth rate. We anticipate that effective tumor cell targeting of hDM will result in localized tumor cytotoxicity *in vivo*. Our findings should provide important insights into approaches for the development of superior all human ADEPT.

## Competing interests

The authors declare that they have no competing interests.

## Authors' contributions

SA designed and carried out all the experiments, and drafted the manuscript. TO and AMW participated in the design of the study. SLM envisioned the overall study and drafted the manuscript. All authors read and approved the manuscript.
